# Genetic variation of *SORBS1* gene is associated with glucose homeostasis and age at onset of diabetes: A SAPPHIRe Cohort Study

**DOI:** 10.1038/s41598-018-28891-z

**Published:** 2018-07-12

**Authors:** Tien-Jyun Chang, Wen-Chang Wang, Chao A. Hsiung, Chih-Tsueng He, Ming-Wei Lin, Wayne Huey-Herng Sheu, Yi-Cheng Chang, Tom Quertermous, Yii-Der Ida Chen, Jerome I. Rotter, Lee-Ming Chuang, The SAPPHIRe Study Group, Chii-Min Hwu, Yi-Jen Hung, Wen-Jane Lee, I-Te Lee

**Affiliations:** 1 Department of Internal Medicine, National Taiwan University Hospital, Taipei, Taiwan; 2 The Ph.D. Program for Translational Medicine, College of Medical Science and Technology, Taipei Medical University, Taipei, Taiwan; 3 Division of Biostatistics and Bioinformatics, Institute of Population Health Sciences, National Health Research Institutes, Zhunan, Taiwan; 4 Department of Endocrinology and Metabolism, Tri-Service General Hospital, Taipei, Taiwan; 5 Institute of Public Health, National Yang-Ming University, Taipei, Taiwan; 6 Department of Medical Research & Education, Taipei Veterans General Hospital, Taipei, Taiwan; 7 Department of Endocrinology and Metabolism, Taichung Veterans General Hospital, Taichung, Taiwan; 8 School of Medicine, National Yang-Ming University, Taipei, Taiwan; 9 School of Medicine, National Defense Medical Center, Taipei, Taiwan; 10 Graduate Institute of Medical Genomics and Proteomics, National Taiwan University Medical College, Taipei, Taiwan; 11 Institute of Biomedical Science, Academia Sinica, Taipei, Taiwan; 12 Division of Cardiovascular Medicine, Falk CVRC, Stanford University School of Medicine, Stanford, CA USA; 13 Institute for Translational Genomics and Population Sciences, Los Angeles Biomedical Research Institute at Harbor-UCLA Medical Center, Torrance, CA USA; 14 Division of Genomic Outcomes, Departments of Pediatrics and Medicine, Harbor-UCLA Medical Center, Torrance, CA USA; 15 Institute of Epidemiology and Preventive Medicine, College of Public Health, National Taiwan University, Taipei, Taiwan; 16 Section of Endocrinology and Metabolism, Taipei Veterans General Hospital, Taipei, Taiwan; 17 Faculty of Medicine, National Yang-Ming University School of Medicine, Taipei, Taiwan; 18 Department of Medical Research, Taichung Veterans General Hospital, Taichung, Taiwan; 19 Department of Social Work, Tunghai University, Taichung, Taiwan; 20 School of Medicine, Chung Shan Medical University, Taichung, Taiwan

## Abstract

The *SORBS1* gene plays an important role in insulin signaling. We aimed to examine whether common single-nucleotide polymorphisms (SNPs) of *SORBS1* are associated with prevalence and incidence of diabetes, age at onset of diabetes, and the related traits of glucose homeostasis. A total of 1135 siblings from 492 ethnic Chinese families were recruited at baseline, and 630 were followed up for 5.19 ± 0.96 years. Nine SNPs including rs7081076, rs2281939, rs3818540, rs2274490, rs61739184, rs726176, rs2296966, rs17849148, and rs3193970 were genotyped and examined. To deal with correlated data of subjects within the same families, the generalized estimating equations approach was applied throughout all association analyses. The *GG* genotype of rs2281939 was associated with a higher risk of diabetes at baseline, an earlier onset of diabetes, and higher steady-state plasma glucose levels in the modified insulin suppression test. The minor allele *T* of rs2296966 was associated with higher prevalence and incidence of diabetes, an earlier onset of diabetes, and higher 2-h glucose during oral glucose tolerance test. These two SNPs revealed independent associations with age of diabetes onset as well as risk of diabetes at baseline. These findings supported that *SORBS1* gene participates in the pathogenesis of diabetes.

## Introduction

Insulin resistance and defects in the secretion of insulin by pancreatic beta cells for the maintenance of glucose homeostasis are the main pathogenetic factors in the development of type 2 diabetes (T2DM)^1^. In addition, both genetic and environmental factors contribute to risk of developing T2DM^2^, with the estimated heritability ranges between 20% and 80%^3^. More than 80 genetic loci have been identified for T2DM using a genome-wide association (GWAS) approach in the past decade^4–6^. However, these newly identified genetic loci of T2DM account for only 5–10% of its heritability^7^; a large proportion of heritability is as yet unexplained and requires further investigation.

We previously cloned a human gene containing a sorbin homology domain and 3 SH3 domains in the C-terminal region, termed *SORBS1* [OMIM 605264, GenBank accession No. AF136380 and AF136381]; this is a human homologue of c-Cbl-associated protein (CAP)^8^. Insulin stimulates phosphorylation of c-Cbl, leading to translocation to a lipid raft domain of the plasma membrane through dissociation of c-Cbl-CAP complex from the insulin receptor, resulting in the translocation of the vesicles with glucose transporter 4 (GLUT4), from cytoplasm to the plasma membrane^9^. Therefore, *SORBS1* is an important adaptor protein in the signaling pathway of insulin-stimulated glucose uptake in the mouse^9^. A previous case-control study showed a positive association of the T228A polymorphism with insulin resistance, obesity and T2DM^10^. The aim of this study was to exam the result of previous study and explore whether novel genetic variants of *SORBS1* are associated with parameters of glucose homeostasis, prevalence of diabetes mellitus (DM), and age at onset of DM in a cohort of ethnic Chinese family members from the Stanford Asia-Pacific Program for Hypertension and Insulin Resistance (SAPPHIRe) study with 5 years of follow-up.

## Results

### Demographic and anthropometric characteristics of study subjects at baseline

A total of 1135 siblings from 492 ethnic Chinese families were recruited at baseline, and 630 were followed up for 5.19 ± 0.96 years. Their mean age was 49.49 ± 8.15 years, and 542 (47.75%) participants were male. In all, 783 (68.99%) subjects had hypertension at baseline, and 25 (12.38%) developed hypertension during follow-up; 146 (12.86%) subjects had DM at baseline, and 118 (21.38%) developed DM during follow-up. The mean body mass index (BMI) was 25.35 ± 3.41 kg/m^2^. The mean fasting glucose and mean fasting insulin concentration were 5.13 ± 0.93 mmol/l and 53.54 ± 35.57 pmol/l, respectively. The mean 2-h glucose and 2-h insulin concentration during OGTT were 7.90 ± 2.75 mmol/l and 474.49 ± 435.37 pmol/l, respectively. The average SSPG was 9.98 ± 4.16 mmol/l, and the average HOMA-IR, HOMA-beta, and HOMA-S were 1.82 ± 1.40, 101.81 ± 232.49, and 0.87 ± 0.67, respectively (Table 1).

**Table 1:** Demographic and anthropometric characteristics of study subjects at baseline.

Variable, unit	N	Mean ± SD / n (%)
Age, year	1135	49.49 ± 8.15
Male	1135	542 (47.75%)
Hypertension at baseline	1135	783 (68.99%)
Incident hypertension during follow-up	202	25 (12.38%)
Diabetes at baseline	1135	146 (12.86%)
Incident diabetes during follow-up	552	118 (21.52%)
BMI, kg/m^2^	1134	25.35 ± 3.41
Fasting insulin, pmol/l	1131	53.54 ± 35.57
2-h insulin, pmol/l	1097	474.49 ± 435.37
Fasting glucose, mmol/l	1135	5.13 ± 0.93
2-h glucose, mmol/l	1081	7.90 ± 2.75
SSPG, mmol/l	343	9.98 ± 4.16
HOMA-IR	1131	1.82 ± 1.40
HOMA-beta	1131	101.81 ± 232.49
HOMA-S	1131	0.87 ± 0.67

*N*, number of subjects having available data; SD, standard deviation.

### Successful rate of genotyping

The information of 9 tag SNPs of *SORBS1* gene was shown at Table 2. As described in Methods, genotyping of rs2281939 was applied to 938 subjects; among which, 886 obtained available genotypes (successful rate: 94.5%). For other eight SNPs, the successful rate of genotyping ranges from 92.9 to 99.5%, with a median of 98.2%. Un-typed or failed genotypes of rs2281939 and failed genotypes of other SNPs were inferred by implementing HAPLORE^11^ and MERLIN^12^. Our association analyses were based on lab-typed and inferred genotype data of 1135 subjects.

**Table 2:** *SORBS1* SNPs information.

SNP	SNP name	Relative position^a,b^ (bp)	Chromosome position^b^ (bp)	Location^b^	Major allele/ Minor allele	MAF	HWE p-value
1	rs7081076	146635	97164527	exon 8	G/T	0.08	0.05
2	rs2281939	146820	97164342	exon 8	A/G	0.11	0.24
3	rs3818540	155361	97155801	intron 10	C/T	0.38	0.48
4	rs2274490	179649	97131513	exon 15	T/C	0.37	0.88
5	rs61739184	179685	97131477	exon 15	C/T	0.07	0.98
6	rs726176	215007	97096155	exon 19	G/A	0.36	0.77
7	rs2296966	246748	97064414	exon 25, 3′UTR	C/T	0.34	1.00
8	rs17849148	247043	97064119	exon 25, 3′UTR	C/T	0.27	0.51
9	rs3193970	249164	97061998	exon 25, 3′UTR	A/G	0.37	0.46

MAF, minor allele frequency; HWE, Hardy-Weinberg equilibrium.

^a^Genomic position relative to transcription start site.

^b^SNP position information is according to NM_015385.

### Association with risk of DM at baseline and incidence of DM during follow-up

The association of minor allele of each SNP with DM risk at baseline and the incidence of DM during follow-up were examined under additive, dominant, and recessive models. As shown in Table 3, significant associations were revealed by rs2281939 and rs2296966. Specifically, subjects carrying the *GG* genotype of rs2281939 had a significantly higher risk of having DM at baseline (odds ratio (O.R.): 4.36, 95% C.I.: 1.11–17.16, *p* = 0.035, *q* = 0.17) compared with other subjects. Association between minor allele *T* of rs2296966 and DM risk at baseline was observed under an additive model (O.R.: 1.33, 95% C.I.: 1.02–1.73, *p* = 0.035, *q* = 0.17). Furthermore, subjects carrying *TT* genotype of rs2296966 had a higher incidence of DM during follow-up (O.R.: 1.93, 95% C.I.: 1.1–3.4, *p* = 0.023, *q* = 0.17).

**Table 3:** Association of the minor alleles of *SORBS1* SNPs with risk of DM at baseline and incidence of DM during follow-up.

SNP	Model	Risk of DM at baseline		Incidence of DM during follow-up	
		O.R. (95% C.I.)	*p*-value (*q*-value)^a^	O.R. (95% C.I.)	*p*-value (*q*-value)^a^
rs7081076	ADD	0.93 (0.58, 1.51)	0.78	0.90 (0.50, 1.63)	0.73
	DOM				
	REC				
rs2281939	ADD	1.09 (0.68, 1.75)	0.71	0.69 (0.39, 1.23)	0.21
	DOM	1.01 (0.64, 1.60)	0.96	0.70 (0.39, 1.25)	0.22
	REC	4.36 (1.11, 17.16)	**0.035** (0.17)		
rs3818540	ADD	1.21 (0.93, 1.57)	0.15	1.21 (0.86, 1.69)	0.27
	DOM	1.21 (0.85, 1.72)	0.29	1.15 (0.71, 1.84)	0.57
	REC	1.42 (0.86, 2.33)	0.17	1.52 (0.85, 2.73)	0.16
rs2274490	ADD	0.97 (0.75, 1.25)	0.79	1.08 (0.78, 1.49)	0.65
	DOM	0.93 (0.64, 1.36)	0.71	1.05 (0.68, 1.63)	0.82
	REC	1.00 (0.63, 1.60)	0.98	1.20 (0.65, 2.22)	0.56
rs61739184	ADD	0.97 (0.59, 1.61)	0.91	1.17 (0.65, 2.09)	0.60
	DOM	0.93 (0.57, 1.55)	0.79		
	REC	3.95 (0.66, 23.64)	0.13		
rs726176	ADD	0.85 (0.66, 1.1)	0.23	1.00 (0.74, 1.36)	0.99
	DOM	0.88 (0.61, 1.28)	0.51	0.86 (0.56, 1.30)	0.47
	REC	0.66 (0.38, 1.17)	0.15	1.32 (0.73, 2.39)	0.35
rs2296966	ADD	1.33 (1.02, 1.73)	**0.035** (0.17)	1.38 (1.00, 1.90)	0.051
	DOM	1.42 (0.98, 2.05)	0.065	1.33 (0.84, 2.11)	0.22
	REC	1.51 (0.89, 2.55)	0.13	1.93 (1.10, 3.40)	**0.023** (0.17)
rs17849148	ADD	0.82 (0.62, 1.09)	0.17	0.85 (0.60, 1.20)	0.35
	DOM	0.80 (0.56, 1.14)	0.22	0.81 (0.52, 1.24)	0.33
	REC	0.74 (0.39, 1.4)	0.35	0.88 (0.39, 1.98)	0.76
rs3193970	ADD	0.86 (0.67, 1.11)	0.25	0.93 (0.68, 1.28)	0.67
	DOM	0.84 (0.58, 1.21)	0.34	0.79 (0.52, 1.20)	0.27
	REC	0.79 (0.47, 1.34)	0.38	1.19 (0.65, 2.16)	0.57

ADD, additive model; DOM, dominant model; REC, recessive model; O.R., odds ratio; C.I., confidence interval.

^a^*P*-value < 0.05 was shown in bold and *q*-value was shown when *p*-value < 0.05.

### Association with overall risk of DM and age of DM onset

Since rs2281939 and rs2296966 showed significant associations with risk of DM at baseline/incidence of DM during follow-up, we further investigated whether they were associated with overall risk of DM and age of DM onset. As shown in Table 4, rs2281939 was only significantly associated with age of DM onset under a recessive genetic model. Subjects carrying the *GG* genotypes of rs2281939 had a younger age at onset of DM (hazard ratio (H.R.): 4.31, 95% C.I.: 2.19–8.47, *p* = 0.000022, *q* = 0.0014) compared with other subjects. On the other hand, the minor allele *T* of rs2296966 was associated with not only a younger age of DM onset but also a higher overall DM risk. Although the associations of rs2296966 with these two phenotypes were significant under all genetic models, the smallest p-values were consistently observed under an additive model. Specifically, when carrying an additional minor allele *T* of rs2296966, the estimated O.R. for overall DM risk was 1.42 (95% C.I.: 1.14–1.77, *p* = 0.0020, *q* = 0.064) and the H.R. for age of DM onset was 1.30 (95% C.I.:1.07–1.57, *p* = 0.0079, *q* = 0.084). Based on the above results, we inferred that the inheritance model for rs2281939 is recessive and for rs2296966 is additive, respectively.

**Table 4:** Association of the minor alleles of rs2281939 and rs2296966 with overall risk of DM and age of DM onset.

SNP	Genotype (*n*)	Model	Overall risk of DM		Age of DM onset		
			O.R. (95% C.I.)	*p*-value (*q*-value)^a^	H.R. (95% C.I.)	*p*-value (*q*-value)^a^	*p*-value of Schoenfeld residuals test
rs2281939	*AA* (869)	ADD	0.82 (0.55, 1.20)	0.30	0.93 (0.65, 1.35)	0.71	0.87
	*AG* (256)	DOM	0.78 (0.53, 1.15)	0.21	0.88 (0.62, 1.26)	0.50	0.71
	*GG* (10)	REC	1.73 (0.42, 7.22)	0.45	4.31 (2.19, 8.47)	**0.000022** (0.0014)	0.38
rs2296966	*CC* (514)	ADD	1.42 (1.14, 1.77)	**0.0020** (0.064)	1.30 (1.07, 1.57)	**0.0079** (0.084)	0.70
	*CT* (486)	DOM	1.42 (1.04, 1.93)	**0.026** (0.17)	1.36 (1.04, 1.78)	**0.024** (0.17)	0.90
	*TT* (135)	REC	1.89 (1.20, 2.89)	**0.0032** (0.068)	1.50 (1.04, 2.16)	**0.031** (0.17)	0.36

ADD, additive model; DOM, dominant model; REC, recessive model; O.R., odds ratio; C.I., confidence interval; H.R., hazard ratio.

^a^*P*-value < 0.05 was shown in bold and *q*-value was shown when* p*-value < 0.05.

### Association with DM-related quantitative traits

Using the results shown in Table 4, we analyzed the association of minor alleles of rs2281939 under a recessive model and rs2296966 under an additive model with various quantitative traits. Subjects carrying the *GG* genotypes of rs2281939 had a higher SSPG (*GG*: 13.34 ± 2.98 mmol/l, *AA*/*AG*: 9.93 ± 4.16 mmol/l; *β* = 3.01, 95% C.I.: 0.81–5.21, *p* = 0.0072, *q* = 0.084, Table 5) and a lower BMI (*GG*: 23.27 ± 2.04 kg/m^2^, *AA*/*AG*: 25.37 ± 3.42 kg/m^2^; *β* = −1.80, 95% C.I.: −3.32– −0.27, *p* = 0.021, *q* = 0.17, Table 5) at baseline. Furthermore, the 2-h glucose levels during OGTT increased along with the number of *T* allele of rs2296966 (*CC*: 7.59 ± 2.61 mmol/l, *CT*: 7.99 ± 2.71 mmol/l, *TT*: 8.17 ± 3.11 mmol/l; *β* = 0.36, 95% C.I.: 0.098–0.63, *p* = 0.0073, *q* = 0.084, Table 5).

**Table 5:** Association of the minor alleles of rs2281939 and rs2296966 with quantitative traits.

Trait	Association of rs2281939 under recessive model			Association of rs2296966 under additive model		
	*β*	95% C.I.	*p*-value (*q*-value)^a^	*β*	95% C.I.	*p*-value (*q*-value)^a^
Fasting insulin	7.35	(−6.15, 20.85)	0.29	0.16	(−2.47, 2.78)	0.91
2-h insulin	126.55	(−291.36, 544.46)	0.55	7.98	(−29.08, 45.05)	0.67
Fasting glucose	0.027	(−0.23, 0.29)	0.84	0.050	(−0.039, 0.14)	0.27
2-h glucose	0.34	(−1.60, 2.27)	0.73	0.36	(0.098, 0.63)	**0.0073** (0.084)
HOMA-IR	0.30	(−0.19, 0.80)	0.23	0.025	(−0.081, 0.13)	0.65
HOMA-beta	−5.37	(−31.76, 21.01)	0.69	−4.35	(−19.38, 10.67)	0.57
HOMA-S	−0.29	(−0.59, 0.01)	0.059	−0.00018	(−0.058, 0.060)	0.995
SSPG	3.01	(0.81, 5.21)	**0.0072** (0.084)	−0.18	(−0.71, 0.36)	0.51
BMI	−1.80	(−3.32, −0.27)	**0.021** (0.17)	0.015	(−0.27, 0.30)	0.92

C.I., confidence interval; SSPG, steady state plasma glucose between 150 and 180 minutes during modified insulin suppression test.

^a^*P*-value < 0.05 was shown in bold and *q*-value was shown when *p*-value < 0.05.

### Rs2281939 and rs2296966 are independently associated with age at onset of DM

To investigate whether rs2281939 and rs2296966 are independently associated with age at onset of DM, we re-examined the associations by incorporating these 2 SNPs into the model simultaneously. As shown in Table 6, compared with the associations observed in 1-SNP models, the respective associations of these two SNPs were strengthened in the 2-SNP model (H.R. for rs2281939: 5.42, 95% C.I.: 2.73–10.78, *p* = 0.0000014; H.R. for rs2296966: 1.32, 95% C.I.: 1.09–1.59, *p* = 0.0043), which implied that rs2281939 and rs2296966 were independently associated with age at onset of DM. Similarly, as shown in Supplementary Table S1, these two SNPs were also independently associated with risk of DM at baseline. The estimated Dʹ of linkage disequilibrium (LD) between rs2281939 and rs2296966 was 0.58.

**Table 6:** Rs2281939 and rs2296966 are independently associated with age of DM onset.

Model	rs2281939			rs2296966		
	H.R.	95% C.I.	*p*-value^a^	H.R.	95% C.I.	*p*-value^a^
1-SNP model: considering rs2281939 alone	4.31	(2.19, 8.47)	**0.000022**			
1-SNP model: considering rs2296966 alone				1.30	(1.07, 1.57)	**0.0079**
2-SNP model: considering rs2281939 & rs2296966 simultaneously	5.42	(2.73, 10.78)	**0.0000014**	1.32	(1.09, 1.59)	**0.0043**

H.R., hazard ratio; C.I., confidence interval.

^a^*P*-value < 0.05 was shown in bold.

### Re-examining associations of rs2281939 using lab-typed genotypes

Because only 886 subjects had lab-typed genotypes of rs2281939, we re-examined above significant findings of rs2281939 by using these subjects. Results of the re-examination were consistent with original findings (Supplementary Table S2).

## Discussion

The main finding of this study is that two common SNPs within *SORBS1* gene, rs2281939 and rs2296966, were independently associated with age at onset of DM in a Han Chinese population. The incidence of T2DM in young adults has been increasing worldwide in recent decades^13–15^. Fasting glucose, 2-h glucose during OGTT, HDL cholesterol and BMI have been reported to be risk factors for early onset of T2DM in children, and in American Indian adolescents^16^. More and more studies have examined the influence of genetic variants on the age at diagnosis of T2DM^17–25^. To the best of our knowledge, this is the first study on the association of genetic variants of *SORBS1* with age at onset of DM. In addition, we found some associations of *SORBS1* SNPs with prevalence and incidence of DM as well as some quantitative traits of glucose homeostasis.

It has been reported that *SORBS1* is an important adaptor protein in the signaling pathway of insulin-stimulated glucose uptake in the mouse^9^. Deletion of the bone marrow-specific *Cap* gene has also been reported to protect against high fat diet-induced insulin resistance^26^. It has also been reported that treatment with an insulin sensitizer, thiazolidinedione, may regulate expression of the *CAP* gene in insulin-sensitive tissues^27^.

In this study, though subjects carrying the *GG* genotype of rs2281939 (T228A polymorphism) of *SORBS1* had lower BMI, they were more insulin-resistant with higher SSPG (Table 5), which may explain the higher risk of DM and earlier age at onset of DM in subjects with the *GG* genotype of rs2281939 (Tables 3 & 4). Although the finding related to BMI was similar to a previous study^10^, the association between rs2281939 and DM risk observed in this study was not consistent with previous studies^10^. Lin *et al*. reported that subjects with *AG* or *GG* genotypes of rs2281939 had lower risk of obesity and T2DM and lower BMI^10^. In the DIAbetes Genetics Replication and Meta-analysis (DIAGRAM) Consortium (GWAS of T2DM, http://www.diagram-consortium.org/), the *A* allele is associated with higher risk of T2DM (O.R. 1.15, 95% C.I. 1.01–1.31, *p* = 0.035, searched on 3.18.2018). The discrepancy between the current and previous studies could be explained, at least partially, by the study populations and the genetic models used. First, due to the study design of SAPPHIRe, about 70% of the study population had hypertension at baseline, which is quite different from the study populations used in Lin *et al*.^10^ and in the DIAGRAM Consortium^28^. Second, all the additive, dominant, and recessive genetic models were considered in the current study and significant associations of rs2281939 with baseline DM risk and age at onset of DM were observed uniformly under a recessive model. However, the association under a recessive model was not examined by the previous studies. Specifically, to investigate the association with DM risk, Lin *et al*. adopted a dominant genetic model^10^, while an additive genetic model was used in the GWAS of DIAGRAM^28^. Nevertheless, it should be noted that, due to the small number of subjects carrying *GG* genotype of rs2281939 in the current study (*n* = 10, Table 4), the observed association of rs2281939 under a recessive model need to be confirmed by further studies with larger samples in the future.

In this study, we found that minor allele of rs2296966 was associated with a higher risk of DM (Tables 3 and 4), a higher incidence of DM during follow-up (Table 3), a younger age of DM onset (Table 4), and a higher level of 2-h glucose during OGTT (Table 5). These associations were observed mainly under an additive genetic model. From the Type 2 Diabetes Knowledge Portal (http://www.type2diabetesgenetics.org/, searched on 3.26.2018), we found that nominally significant associations between rs2296966 and risk of DM were observed in three previous studies, including BioMe AMP T2D GWAS (OR = 0.838, p = 0.036), GWAS SIGMA (OR = 1.13, p = 0.0232), and GoT2D WGS (OR = 1.25, p = 0.0483), from different populations. Among which, two studies also showed that minor allele *T* was associated with higher risk of T2DM. Therefore, this study not only replicated an association between rs2296966 and DM risk but also demonstrated that this association may exist in multiple ethnic populations.

Compared with the European populations, there is a tendency to develop DM at a younger age in the Asian populations^29^. In addition to lifestyle and environmental factors, this phenomenon could partially result from genetic factors. In this study, we found that minor allele of rs2296966 was associated with a younger age of DM onset. According to data from 1000 Genomes Project phase 3 (https://www.1000genomes.org/)^30^, the minor allele frequencies (MAF) of rs2296966 in the Asian and European populations are 0.392 and 0.067, respectively. Since MAF of rs2296966 in Asians is much higher than that in Europeans, the earlier onset of DM in Asians^31^ might be explained partially by the observed association of rs2296966 with younger age of DM onset found in this study and the large difference in MAFs between Asian and European populations.

Although the genetic variants of rs2281939 and rs2296966 were both associated with a younger age of DM onset, the result of 2-SNP model analysis showed that the associations revealed by these two SNPs were independent (Table 6). Similar result was obtained from 2-SNP model analysis for risk of DM at baseline (Supplementary Table S1). In addition, only rs2281939 was associated with SSPG and BMI and only rs2296966 was associated with incidence of DM during follow-up and 2-h glucose during OGTT. These observations indicated that these two SNPs affect DM-associated phenotypes in different ways. We note that the estimated D′ of LD between rs2281939 and rs2296966 was 0.58, which showed a low extent of LD between these two SNPs. The genetic polymorphism of rs2281939 confers a substitution of alanine for threonine in codon 228, which may change the function of *SORBS1* and influence the insulin signaling pathway. Rs2296966 is located in 3′UTR of *SORBS1*. According to the miRNASNP database^32^ (http://bioinfo.life.hust.edu.cn/miRNASNP2/, searched on 4.10.2018), we found that the SNP of rs2296966 is related to the potential binding sites of two microRNAs. Specifically, *T* allele of rs2296966 will cause gain of the target site of hsa-miR-526b-5p and loss of target site of hsa-miR-635. Therefore, the substitution of *T* allele for *C* allele may change the binding site to specific microRNA, which leads to alter protein expression or mRNA stability^33^ of *SORBS1* gene. This may explain why the two SNPs affect the phenotypes in slightly different ways.

There are several strengths to this study. First, to the best of our knowledge, this is the first *SORBS1* genetic study investigating comprehensive DM-related traits. We examined the association between *SORBS1* SNPs and age at onset of diabetes, in addition to prevalence of diabetes in this study. We also analyzed the association of *SORBS1* SNPs with multiple metabolic traits and incidence of DM in this study. Second, we didn’t assume specific genetic inheritance in the association analysis. Instead, we analyzed the association of SNPs with risk of DM at baseline, incidence of DM during follow-up, overall risk of DM and age of DM onset in this study under additive, dominant, and recessive model, respectively. Third, instead of using a stringent threshold for *p*-values required by Bonferroni correction, we implemented a flexible false discovery rate (FDR)-based approach to deal with the problem of multiple testing. For each association test, in addition to p-value, we also reported a *q*-value, which is a measure of significance in terms of FDR^34^. Then, by determining a threshold for *q*-values to declare significant findings in multiple comparisons, we can control the expected proportion that declared associations are truly null. In this study, we used a threshold of 0.2, which was also adopted by recent studies^35,36^. Using such a less stringent threshold should enable us to find moderate associations, while controlling a reasonable FDR. However, there is a limitation in this study. According to analysis of the 2000–2009 nationwide health insurance database in Taiwan, the incidence of diabetes at the age group 20–79 y/o in 2008 is 1.16%^37^. In this study, the incidence of diabetes during follow up for 5.19 ± 0.96 years is 21.38% (4.12%/yr), which is much higher than the incidence of Taiwanese national database. The probands recruited in this study had hypertension, and the prevalence rate of hypertension at baseline is high (68.99%). Several studies supported that impaired insulin signaling involved in the pathogenesis of essential hypertension^38,39^. Hence, the higher incidence of diabetes in this study than that in the normal population may be partially due to the higher prevalence of hypertension at baseline. Therefore, the association obtained from this study may not be extrapolated to non-hypertensive populations.

In conclusion, we reported the associations of 2 common SNPs of *SORBS1* with prevalence and incidence of DM, age at onset of DM, and some quantitative traits of glucose homeostasis. These findings, together with earlier observations in different ethnic groups, support an involvement of the *SORBS1* gene in the pathogenesis and clinical phenotypes of DM.

## Methods

### Subjects of the SAPPHIRe study cohort

The Stanford Asia-Pacific Program for Hypertension and Insulin Resistance (SAPPHIRe) was a collaborative family study designed to investigate the genetic determinants of hypertension and insulin resistance in ethnic Chinese and Japanese. The design of SAPPHIRe study recruited both concordant siblings (all siblings with hypertension) and discordant siblings (at least one hypertensive sibling). Index cases were determined as those with age at onset of 35–60 years or those 60 years of age with corroborating record of their hypertension status before age 60 years^40^. In the present study, we just selected siblings from ethnic Chinese nuclear families for genotyping. In the present study, 1135 siblings from 492 ethnic Chinese nuclear families were genotyped. Among which, 630 received follow-up exam approximately 5 years later. The study was approved by Institutional Review Boards at all participating sites, including National Taiwan University Hospital, Taipei Veterans General Hospital, Tri-Service General Hospital, and Taichung Veterans General Hospital. The methods were carried out in accordance with the relevant guidelines and regulations. Written informed consent was obtained from all subjects prior to their enrollment in the study.

### Phenotyping

Body height (BH), body weight (BW), and BMI [BW (kg)/BH^2^ (m^2^)] were measured at 8 AM, after an overnight fast for 8–10 hrs. Each subject received a 75-g oral glucose tolerance test (OGTT) after the anthropometric measurements. Fasting blood samples were obtained for measuring plasma glucose and insulin concentration. Then, the subjects drank 75-g glucose in 300 ml water within 5 min. Blood samples were taken for plasma glucose and insulin 1- and 2-h after glucose loading^40^. Plasma glucose and insulin levels were measured as described previously^40^. Insulin resistance index (HOMA-IR), beta cell function index (HOMA-beta), and sensitivity index (HOMA-S) derived from the homeostasis model were calculated as in the previous study^40^. DM was defined as fasting plasma glucose ≥7.0 mmol/l (126 mg/dl), or 2-h glucose in OGTT ≥11.1 mmol/l (200 mg/dl), or use of at least 1 antidiabetic agent, or self-reported DM history at baseline or during follow-up. All subjects diagnosed as having DM during follow-up were classified as having T2DM by endocrine specialists based on their BMI, family history of DM, age, and the fact that no one had diabetic ketoacidosis. One subject developed type 1 DM during follow-up was excluded. Hypertension was defined as systolic blood pressure >140 mmHg, diastolic blood pressure >90 mmHg, or use of at least one medication to control high blood pressure.

We quantified insulin sensitivity in a subset of 344 participants in the SAPPHIRe cohort at baseline using a modified insulin suppression test^41^. In brief, after overnight fasting, a venous catheter was placed in each of the subjects’ arms. One arm was used for the infusion of octreotide (0.5 μg/min preceded by a 25 μg bolus), insulin (25 mU⋅m^2^/min), and glucose (40 mg⋅m^2^/min). Plasma glucose levels were measured between 150 and 180 min after the infusion. The mean of the glucose levels was termed steady-state plasma glucose (SSPG); this provided a direct measure of the ability of insulin to mediate the disposal of infused glucose^41^.

### Selection of tag SNPs and genotyping

Initially, eight tag SNPs were selected from the HapMap Chinese in Beijing (CHB) data bank (http://www.hapmap.org) using the Tagger program in Haploview 4.1 (http://www.broad.mit.edu/mpg/haploview/)^42^, with a minor allele frequency threshold of 5% and a *r*^2^ of 0.8 (Table 2). Total genomic DNA was purified from peripheral blood leukocytes using a Puregene DNA extraction kit (Minneapolis, MN, USA), according to the manufacturer’s protocol. Genotyping was performed using Applied Biosystems SNPlex assays.

Later, rs2281939, which has been shown to be associated with insulin resistance, obesity and T2DM^10^ was also selected in this study. Among the 1135 study subjects, the genotyping of rs2281939 was applied only to a subset of 938 subjects. Un-typed or failed genotypes were inferred by implementing software HAPLORE^11^ and MERLIN^12^. All SNPs were in Hardy–Weinberg equilibrium in the controls (all p > 0.01), as determined by the Haploview program^42^.

All methods were carried out in accordance with the approved guidelines, and all experimental protocols were approved by committees at National Taiwan University Hospital, Taipei Veterans General Hospital, Taichung Veterans General Hospital, and Tri-Service General Hospital.

### Statistical analysis

Continuous variables were presented as mean values ± standard deviation (SD), and binary variables were presented as count (percentage) of a specific category. The associations of individual *SORBS1* SNPs with the risk of DM at baseline and the incidence of DM during follow-up under different genetic models were assessed by implementing multiple logistic regression analyses and were adjusted for age, gender, BMI, and hypertension status. Furthermore, we also investigated the associations with overall risk of DM, which were adjusted for birth year, gender, BMI, and hypertension status. The multiple linear regression analyses were used to examine the associations of SNPs with quantitative traits including BMI, fasting glucose/insulin, 2-h glucose/insulin during OGTT, HOMA-IR, HOMA-B, HOMA-S, and SSPG. For BMI, the covariates used for adjustment included age, gender, and hypertension status. For other quantitative traits, the same covariates as well as BMI were used for adjustment. Since the subjects analyzed in this study were siblings from nuclear families, a generalized estimating equations (GEE) approach^43^ was applied throughout the logistic and linear regression analyses to deal with the correlated data within the same families. The GEE approach is an extension of the quasi-likelihood method which takes within-cluster correlations into account by using a set of equations and sums these equations over all clusters to obtain the population-averaged estimates of the parameters^44^. The GEE analyses were implemented using IBM SPSS version 19.0.

We used a Cox proportional hazard model^45^ to test for associations between specific *SORBS1* SNPs and age at onset of DM, which were adjusted for birth year, gender, BMI, and hypertension status. Cox regression analysis was performed using the R package “survival” [version 2.38–3, downloaded from The Comprehensive R Archive Network (CRAN)], and the robust sandwich variance estimator^46^ was used to deal with the within-family correlation of age at onset of DM. Furthermore, the Schoenfeld residuals test was performed to examine whether our data were fit for the proportional hazards assumption essential for Cox regression analysis^47^.

To deal with the problem of multiple testing, a measure of significance in terms of FDR, called *q*-value, was calculated by QVALUE software^34^ for each association test. In this study, an association with a *q*-value less than 0.2 was considered significant under multiple comparisons. With this threshold of *q*-value, the expected proportion of false positive findings among the declared associations is 20%.

### Data availability

The datasets generated during and/or analysed during the current study are available from the corresponding author on reasonable request.

## Electronic supplementary material

Supplementary Table 1 & 2
