# Supplementary material for: Genetic variation of SORBS1 gene is associated with glucose homeostasis and age at onset of diabetes: A SAPPHIRe Cohort Study
Source: Sci Rep. 2018 Jul 12;8:10574. doi: 10.1038/s41598-018-28891-z (PMC6043583; doi:10.1038/s41598-018-28891-z)
Supplement: Supplementary file 1 — Supplementary Table 1 & 2 [file 41598_2018_28891_MOESM1_ESM.docx]

Supplementary Information

**Genetic variation of *SORBS1* gene is associated with glucose homeostasis and age at onset of diabetes: A SAPPHIRe Cohort Study**

Tien-Jyun Chang^1^*, Wen-Chang Wang^2,3^*, Chao A. Hsiung^3^, Chih-Tsueng He^4^, Ming-Wei Lin^5,6^, Wayne Huey-Herng Sheu^7,8,9^, Yi-Cheng Chang^1,10,11^, Tom Quertermous^12^, Yii-Der Ida Chen^13^, Jerome I. Rotter^13,14^, Lee-Ming Chuang^1, 15*^*,* the SAPPHIRe Study Group

^1^ Department of Internal Medicine, National Taiwan University Hospital, Taipei, Taiwan

^2^ The Ph.D. Program for Translational Medicine, College of Medical Science and Technology, Taipei Medical University, Taipei, Taiwan

^3^ Division of Biostatistics and Bioinformatics, Institute of Population Health Sciences, National Health Research Institutes, Zhunan, Taiwan

^4^ Department of Endocrinology and Metabolism, Tri-Service General Hospital, Taipei, Taiwan

^5^ Institute of Public Health, National Yang-Ming University, Taipei, Taiwan

^6^ Department of Medical Research & Education, Taipei Veterans General Hospital, Taipei, Taiwan

^7^ Department of Endocrinology and Metabolism, Taichung Veterans General Hospital, Taichung, Taiwan

^8^ School of Medicine, National Yang-Ming University, Taipei, Taiwan

^9^ School of Medicine, National Defense Medical Center, Taipei, Taiwan

^10^ Graduate Institute of Medical Genomics and Proteomics, National Taiwan University Medical College, Taipei, Taiwan

^11^ Institute of Biomedical Science, Academia Sinica, Taipei, Taiwan

^12^ Division of Cardiovascular Medicine, Falk CVRC, Stanford University School of Medicine, Stanford, CA

^13^ Institute for Translational Genomics and Population Sciences, Los Angeles Biomedical Research Institute at Harbor-UCLA Medical Center, CA

^14^ Division of Genomic Outcomes, Departments of Pediatrics and Medicine, Harbor-UCLA Medical Center, CA

^15^ Institute of Epidemiology and Preventive Medicine, College of Public Health, National Taiwan University, Taipei, Taiwan

The SAPPHIRe Study Group: Chii-Min Hwu^16,17^, Yi-Jen Hung^4,9^, Wen-Jane Lee^18,19^, I-Te Lee^7,8,20^

^16^ Section of Endocrinology and Metabolism, Taipei Veterans General Hospital, Taipei, Taiwan

^17^ Faculty of Medicine, National Yang-Ming University School of Medicine, Taipei, Taiwan

^18^ Department of Medical Research, Taichung Veterans General Hospital, Taichung, Taiwan

^19^ Department of Social Work, Tunghai University, Taichung

^20^ School of Medicine, Chung Shan Medical University, Taichung, Taiwan

**Supplementary Table S1. Rs2281939 and rs2296966 are independently associated with risk of DM at baseline.**

| Model | rs2281939 | | |  | rs2296966 | | |
| --- | --- | --- | --- | --- | --- | --- | --- |
|  | O.R. | (95% C.I.) | *p*-value^a^ |  | O.R. | (95% C.I.) | *p*-value^a^ |
| 1-SNP model: considering rs2281939 alone | 4.36 | (1.11, 17.16) | 0.035 |  |  |  |  |
| 1-SNP model: considering rs2296966 alone |  |  |  |  | 1.33 | (1.02, 1.73) | 0.035 |
| 2-SNP model: considering rs2281939 & rs2296966 simultaneously | 5.68 | (1.47, 21.85) | 0.012 |  | 1.37 | (1.05, 1.78) | 0.019 |

O.R., odds ratio; C.I., confidence interval.

^a^ *P*-value < 0.05 was shown in bold and q-value was shown when p-value < 0.05.

**Supplementary Table S2. Re-examining associations of rs2281939 under recessive model using lab-typed genotypes.**

| Risk of DM at baseline | |  | Age of DM onset | |  | SSPG at baseline | | |  | BMI at baseline | | |
| --- | --- | --- | --- | --- | --- | --- | --- | --- | --- | --- | --- | --- |
| O.R.  (95% C.I.) | *p*-value |  | H.R.  (95% C.I.) | *p*-value |  | Values of different genotypes (mmol/L) | *β*  (95% C.I.) | *p*-value |  | Values of different genotypes (kg/m^2^) | *β*  (95% C.I.) | *p*-value |
| 3.85  (0.90, 16.51) | 0.069 |  | 3.64  (1.59, 8.34) | 0.0023 |  | GG:  13.45 ± 3.43  *AA*/*AG*:  10.12 ± 4.15 | 3.86  (1.63, 6.10) | 0.00071 |  | GG:  23.06 ± 2.08  *AA*/*AG*:  25.35 ± 3.45 | -2.06  (-3.71, -0.40) | 0.015 |

O.R., odds ratio; H.R., hazard ratio; C.I., confidence interval.
